# Supplementary material for: A situational assessment of treatments received for childhood diarrhea in the Federal Republic of Nigeria
Source: PLoS One. 2024 May 22;19(5):e0303963. doi: 10.1371/journal.pone.0303963 (PMC11111079; doi:10.1371/journal.pone.0303963)

PLOS ONE

A situational assessment of treatments received for childhood diarrhea in the Federal Republic of Nigeria

--Manuscript Draft--

**Manuscript Number:**	PONE-D-23-41643
**Article Type:**	Research Article
**Full Title:**	A situational assessment of treatments received for childhood diarrhea in the Federal Republic of Nigeria
**Short Title:**	Treatments received for childhood diarrhea in the Federal Republic of Nigeria
**Corresponding Author:**	Ezra Gayawan<br>Federal University of Technology Akure<br>Akure, NIGERIA
**Keywords:**	diarrhea; treatment seeking; Nigeria; Oral Rehyrdation Therapy
**Abstract:**	We assess progress towards improved case management of childhood diarrhea in Nigeria over a period of targeted health systems reform from 2013 to 2018. Individual and community data from three Demographic and Health Survey rounds are leveraged in a geospatial model designed for stratified estimation by venue of treatment seeking and State. Our analysis reveals a highly regionalised health system undergoing rapid change. Nationally, there have been substantial increases in the proportion of children under 5 years old with diarrhea receiving the recommended oral rehydration therapy after seeking treatment at either a health clinic (0.57 [0.44--0.69; 95\% CrI] in 2008; 0.70 [0.54--0.83] in 2018) or chemist/pharmacy (0.28 [0.17--0.42] in 2008; 0.48 [0.31--0.64] in 2018). Yet State-level variations in venue attendance and performance by venue have conspired to hold the overall proportion receiving this potentially life-saving therapy (0.45 [0.35--0.55] in 2018) to well-below ideal coverage levels. High performing states that have demonstrated significant improvements include Kano, Jigawa and Borno, while under-performing states that have suffered declines in coverage include Kaduna and Taraba. The use of antibiotics is not recommended for mild cases of childhood diarrhea yet remains concerningly high nationally (0.27 [0.19--0.36] in 2018) with negligible variation between venues. Antibiotic use rates are particularly high in Enugu, Kaduna, Taraba, Kano, Niger and Kebbi, yet welcome reductions were identified in Jigawa, Adamawa and Osun. These results support the conclusions of previous studies and build the strength of evidence that urgent action is needed throughout the multi-tiered health system to improve the quality and equity of care for common childhood illnesses in Nigeria.
**Order of Authors:**	Ezra Gayawan
	Ewan Cameron
	Tolu Okitika
	Osafu Egbon
	Peter Gething
**Additional Information:**	
**Question**	**Response**
**Financial Disclosure**<br><br>Enter a financial disclosure statement that describes the sources of funding for the work included in this submission. Review the submission guidelines for detailed requirements. View published research articles from *PLOS ONE* for specific examples.	Yes

Powered by Editorial Manager® and ProduXion Manager® from Aries Systems Corporation

This statement is required for submission and **will appear in the published article** if the submission is accepted. Please make sure it is accurate.<br><br>**Funded studies**<br>Enter a statement with the following details:<br>• Initials of the authors who received each award<br>• Grant numbers awarded to each author<br>• The full name of each funder<br>• URL of each funder website<br>• Did the sponsors or funders play any role in the study design, data collection and analysis, decision to publish, or preparation of the manuscript?<br><br>Did you receive funding for this work?	
Please add funding details.<br>as follow-up to "**Financial Disclosure**<br><br>Enter a financial disclosure statement that describes the sources of funding for the work included in this submission. Review the submission guidelines for detailed requirements. View published research articles from *PLOS ONE* for specific examples.<br><br>This statement is required for submission and **will appear in the published article** if the submission is accepted. Please make sure it is accurate.<br><br>**Funded studies**<br>Enter a statement with the following details:<br>• Initials of the authors who received each award<br>• Grant numbers awarded to each author<br>• The full name of each funder<br>• URL of each funder website<br>• Did the sponsors or funders play any role in the study design, data collection and analysis, decision to publish, or preparation of the manuscript?	Gates Foundation [INV-009390 OPP1197730]<br>Peter Gething was additionally supported through funding provided jointly by Curtin University, the Telethon Trust and the Telethon Kids Institute under project ID RES-61992

Did you receive funding for this work?"	
Please select the country of your main research funder (please select carefully as in some cases this is used in fee calculation).<br>as follow-up to "**Financial Disclosure**<br><br>Enter a financial disclosure statement that describes the sources of funding for the work included in this submission. Review the submission guidelines for detailed requirements. View published research articles from *PLOS ONE* for specific examples.<br><br>This statement is required for submission and **will appear in the published article** if the submission is accepted. Please make sure it is accurate.<br><br>**Funded studies**<br>Enter a statement with the following details:<br>• Initials of the authors who received each award<br>• Grant numbers awarded to each author<br>• The full name of each funder<br>• URL of each funder website<br>• Did the sponsors or funders play any role in the study design, data collection and analysis, decision to publish, or preparation of the manuscript?<br><br>Did you receive funding for this work?"	UNITED STATES - US
**Competing Interests**<br><br>Use the instructions below to enter a competing interest statement for this submission. On behalf of all authors, disclose any competing interests that could be perceived to bias this work—acknowledging all financial support and any other relevant financial or non-financial competing interests.<br><br>This statement is required for submission	The authors have declared that no competing interests exist.

and **will appear in the published article** if the submission is accepted. Please make sure it is accurate and that any funding sources listed in your Funding Information later in the submission form are also declared in your Financial Disclosure statement.<br><br>View published research articles from *PLOS ONE* for specific examples.<br><br>**NO authors have competing interests**<br><br>Enter: *The authors have declared that no competing interests exist.*<br><br>**Authors with competing interests**<br><br>Enter competing interest details beginning with this statement:<br><br>*I have read the journal's policy and the authors of this manuscript have the following competing interests: [insert competing interests here]*<br><br>* typeset	
**Ethics Statement**<br><br>Enter an ethics statement for this submission. This statement is required if the study involved:<br><br>• Human participants<br>• Human specimens or tissue<br>• Vertebrate animals or cephalopods<br>• Vertebrate embryos or tissues<br>• Field research<br><br>Write "N/A" if the submission does not require an ethics statement.<br><br>General guidance is provided below. Consult the submission guidelines for detailed instructions. **Make sure that all information entered here is included in the Methods section of the manuscript.**	N/A

**Format for specific study types**<br><br>**Human Subject Research (involving human participants and/or tissue)**<br>• Give the name of the institutional review board or ethics committee that approved the study<br>• Include the approval number and/or a statement indicating approval of this research<br>• Indicate the form of consent obtained (written/oral) or the reason that consent was not obtained (e.g. the data were analyzed anonymously)<br><br>**Animal Research (involving vertebrate animals, embryos or tissues)**<br>• Provide the name of the Institutional Animal Care and Use Committee (IACUC) or other relevant ethics board that reviewed the study protocol, and indicate whether they approved this research or granted a formal waiver of ethical approval<br>• Include an approval number if one was obtained<br>• If the study involved *non-human primates*, add *additional details* about animal welfare and steps taken to ameliorate suffering<br>• If anesthesia, euthanasia, or any kind of animal sacrifice is part of the study, include briefly which substances and/or methods were applied<br><br>**Field Research**<br><br>Include the following details if this study involves the collection of plant, animal, or other materials from a natural setting:<br>• Field permit number<br>• Name of the institution or relevant body that granted permission	
**Data Availability**<br><br>Authors are required to make all data underlying the findings described fully available, without restriction, and from the time of publication. PLOS allows rare exceptions to address legal and ethical concerns. See the PLOS Data Policy and FAQ for detailed information.	Yes - all data are fully available without restriction

A Data Availability Statement describing where the data can be found is required at submission. Your answers to this question constitute the Data Availability Statement and **will be published in the article**, if accepted.<br><br>**Important:** Stating 'data available on request from the author' is not sufficient. If your data are only available upon request, select 'No' for the first question and explain your exceptional situation in the text box.<br><br>Do the authors confirm that all data underlying the findings described in their manuscript are fully available without restriction?	
**Describe where the data may be found in full sentences. If you are copying our sample text, replace any instances of XXX with the appropriate details.**<br><br>• If the data are **held or will be held in a public repository**, include URLs, accession numbers or DOIs. If this information will only be available after acceptance, indicate this by ticking the box below. For example: *All XXX files are available from the XXX database (accession number(s) XXX, XXX.).*<br>• If the data are all contained **within the manuscript and/or Supporting Information files**, enter the following: *All relevant data are within the manuscript and its Supporting Information files.*<br>• If neither of these applies but you are able to provide **details of access elsewhere**, with or without limitations, please do so. For example:<br><br>*Data cannot be shared publicly because of [XXX]. Data are available from the XXX Institutional Data Access / Ethics Committee (contact via XXX) for researchers who meet the criteria for access to confidential data.*<br><br>*The data underlying the results presented in the study are available from (include the name of the third party*	The data underlying the results presented in the study are available from The DHS Program https://dhsprogram.com/

and contact information or URL).<br>• This text is appropriate if the data are owned by a third party and authors do not have permission to share the data.<br><br>* typeset	
Additional data availability information:	

Click here to access/download;Manuscript;main.pdf

A situational assessment of treatments received for childhood diarrhea in the Federal Republic of Nigeria

Ezra Gayawan[1,2◕*], Ewan Cameron[2,3◕], Tolu Okitika[2] Osafu Augustin Egbon[1,4] Peter Gething[2,3]

1 Department of Statistics, Federal University of Technology, Akure, Nigeria

2 Geospatial Health & Development, Telethon Kids Institute, Nedlands, Western Australia, Australia

3 School of Population Health, Curtin University, Bentley, Western Australia, Australia

4 Department of Statistics, Federal University of São Carlos, São Carlos, Brazil

◕These authors contributed equally to this work. * egayawan@futa.edu.ng

Abstract

We assess progress towards improved case management of childhood diarrhea in Nigeria over a period of targeted health systems reform from 2013 to 2018. Individual and community data from three Demographic and Health Survey rounds are leveraged in a geospatial model designed for stratified estimation by venue of treatment seeking and State. Our analysis reveals a highly regionalised health system undergoing rapid change. Nationally, there have been substantial increases in the proportion of children under 5 years old with diarrhea receiving the recommended oral rehydration therapy after seeking treatment at either a health clinic (0.57 [0.44–0.69; 95% CrI] in 2008; 0.70 [0.54–0.83] in 2018) or chemist/pharmacy (0.28 [0.17–0.42] in 2008; 0.48 [0.31–0.64] in 2018). Yet State-level variations in venue attendance and performance by venue have conspired to hold the overall proportion receiving this potentially life-saving therapy (0.45 [0.35–0.55] in 2018) to well-below ideal coverage levels. High performing states that have demonstrated significant improvements include Kano,

Jigawa and Borno, while under-performing states that have suffered declines in coverage include Kaduna and Taraba. The use of antibiotics is not recommended for mild cases of childhood diarrhea yet remains concerningly high nationally (0.27 [0.19–0.36] in 2018) with negligible variation between venues. Antibiotic use rates are particularly high in Enugu, Kaduna, Taraba, Kano, Niger and Kebbi, yet welcome reductions were identified in Jigawa, Adamawa and Osun. These results support the conclusions of previous studies and build the strength of evidence that urgent action is needed throughout the multi-tiered health system to improve the quality and equity of care for common childhood illnesses in Nigeria.

Author summary

Diarrheal illnesses are amongst the leading causes of childhood mortality in Nigeria. Oral rehydration therapy (preferably administered with zinc supplementation) is a cheap and highly effective treatment recommended by the World Health Organisation. Increasing the proportion of ill children receiving this therapy has been the focus of multiple initiatives at Federal and State level, yet nationally, the mean coverage level remains low. Conversely, antibiotics are not recommended for mild cases of childhood diarrhea but are used at a similar rate, contributing to poor patient outcomes and antibiotic resistance. Community care with oral rehydration therapy, either home made or purchased from a chemist/pharmacy, is recommended for mild cases, yet in many areas, health clinics remain the preferred treatment seeking venue. Our geospatial modelling study combines three rounds of Demographic and Health Surveys to uncover key trends by State and treatment seeking venue. High performing and under-performing areas of the health system are thereby identified; the former provide important case studies of progress while the latter demand urgent action to improve quality of care.

Introduction

Diarrheal illnesses are amongst the leading causes of childhood mortality in Nigeria, accounting for 16% of child deaths [1]. Oral rehydration solution (ORS), a mixture of

clean water, salt and sugar, and preferably administered with zinc supplementation, has been shown to reduce the mean duration of diarrhea (by about 20%), mortality due to diarrhea (by 23%), stool output and the risk of a subsequent episode in the 2 to 3 months after supplementation [2–4]. However, usage of ORS is reported to be low in most low- and middle-income countries [5, 6]. The use of recommended home fluid (RHF) was promoted by the World Health Organization (WHO) in addition to ORS and the combination is referred to as oral rehydration therapy (ORT). As a non-packaged home fluid alternative, RHF can comprise measured sugar and salt added to clean water or other home fluids such as rice water, coconut water, juice or tea [7, 8].

Sustainable Development Goal (SDG) 3.8 is to achieve universal health coverage, including access to essential health care services and access to safe, effective, quality and affordable essential medicines for all. However, there has been slow progress in increasing access to simple and affordable treatments in Nigeria. The proportion of children with diarrhea who received either ORS or RHF increased from 31% to 42% over a ten-year period (2008-2018), while those for whom treatment was sought rose from 42% to 65% [9]. These national figures, however, mask substantial local variations in common with other health indicators in Nigeria [10, 11]. About 3% of the children from Taraba state that had diarrhea in 2018 received ORS and zinc supplements compared with 72% in Nasarawa state [5]. Appropriate and timely care-seeking in formal health facilities would allow for prompt and correct diagnosis, appropriate management and avert complications [12, 13].

Many Nigerian mothers have adequate knowledge of what constitutes diarrhea and could identify episodes of it in their children [14]. But poor knowledge of the use of ORS and zinc supplementation has been identified as a major barrier to improving usage across different parts of Nigeria [1]. There are mothers who hold the myth that diarrhea is a sign that the child is teething, which can negatively influence their health care decision making [1, 15]. A study in eight Nigerian States, and another in Oyo State, identified unavailability, unaffordability and poor awareness as the major barriers to ORS/zinc utilization [13, 16]. Encouragingly, consultation with a health worker, having a secondary or higher level of education and exposure to mass media were reported to encourage the use of ORT for the treatment of diarrhea [17]. The

venue where treatment was sought also substantially affects the chances of receiving ORS or ORS and zinc supplementation across the country [13].

Because it is rarely ~~microbially~~ caused, the use of antibiotics for infectious diarrhea brings little benefit in most cases [18]. The WHO guidelines on the treatment of diarrhea therefore discourages the use of antibiotics for treating non-severe cases, restricting its use to instances of bloody diarrhea and cholera with severe dehydration [19, 20]. Indiscriminate use of antibiotics leads to antibiotic resistance, capable of compromising the treatment of infectious diseases and undermining other advances in health and medicine [19]. Over-use of antibiotics also leads to increased medical costs, longer hospital stays and increases the risk of subsequent diarrhea episodes because of its effects on gut microflora [21]. Notwithstanding, studies have reported abundant use of antibiotics in treating mild cases of diarrhea in various parts of Nigeria [20–23].

We set out to quantify trends by venue in treatment seeking behavior for childhood diarrhea among under-five children according to States of Nigeria. Our objective is to quantify progress in diarrhea treatment in the country over a ten-year period considering the various interventions and programs put in place over the years. For instance, the Essential Childhood Medicines Scale-up Plan (ECMSP) (2012-2015) was contemplated as the country's first national road map for increasing access to life-saving treatments [24]. A program was then implemented by the Clinton Health Access Initiative (CHAI) to align with the proposed strategies and activities under the ECMPS in eight Nigerian states between 2013 and 2017 [13]. We used data from three waves of Nigeria Demographic and Health Surveys (NDHS) conducted in 2008, 2013 and 2018 and adopted a Bayesian geostatistical modeling technique that incorporates the sampling characteristics of the surveys. This allows for more accurate estimates of the treatment seeking behavior at State levels and provides actionable insight for policy makers at and above this adminstrative scale.

Materials and methods

Datasets

Data was compiled from three waves of the NDHS conducted in 2008, 2013 and 2018. Implemented by the Nigeria Population Commission with technical support from the DHS Program, these nationally-representative surveys offer quality assured cross-sectional data on a diverse collection of demographic and health indicators. The standardised methodology employed in each survey facilitates comparative analyses across time and space. A two-stage stratified cluster design was used for data collection. First, enumeration areas (clusters) were selected from a sampling frame created for the 2006 Population and Housing Census of the Federal Republic of Nigeria; and second, households were selected from each cluster by equal probability sampling. A total of 777, 904 and 1,400 clusters were randomly selected for the 2008, 2013 and 2018 surveys, respectively, from which representative samples of 34,070, 40,680 and 42,000 households were constructed. The response rates for the eligible women in each survey, who must be between ages 15 and 49 years, were 97%, 98% and 99%.

Information on child health and interactions with the local health system was collected for children under 5 y/o living in the surveyed households. Data concerning experiences of ill health were collected by questionnaire targeting events in the two weeks prior to the surveys, answered on the child's behalf by the mother or caregiver. A child was identified to have suffered from diarrhea if the child had three or more loose or liquid stools in the same day (on one or more days of the preceding two weeks). Whenever a child is positively identified as having experienced diarrhea, the caregiver was asked if the child was given oral rehydration/ORS or antibiotic pills or syrups. If the child received any treatment, the caregiver was asked to indicate where the treatment was sought. The response variables for our analysis were constructed as binary indicators as for: (i) each of ORS/RHF and antibiotic, indicating whether or not the child was treated with any of these and (ii) each of treatment-seeking class indicating whether or not treatment was sought at a health facility (private or public facility), a chemist/pharmacy or treated at home.

Geostatistical Model

Given the multistage structure employed in gathering the DHS data, a key element in geostatistical modeling is to include the sampling characteristics in the model, as doing otherwise could potentially bias the estimates. We adopt a geostatistical technique for areal data that considers the stratified multistage cluster design used in data collection as proposed by Chen and colleagues [25]. The approach proceeds by first computing the proportion for each location using a designed-based weighted (direct) estimator:

$$p_i^{HT} = \frac{\sum_{c \in s_i} \sum_{k \in s_c} w_{ck} Y_{ck}}{\sum_{c \in s_i} \sum_{k \in s_c} w_{ck}} \quad (1)$$

where $c = 1 \cdots C$ are clusters sampled within strata, and for each cluster c, $k \in s_c$ is the indexes of individuals sampled in each cluster c. The binary outcome Y_{ck} is the quantity whose prevalence is to be estimated, w_{ck} are the sampling weights and s_i is the indices of clusters sampled in area i. To enhance precision, a transformation of this weighted estimate can thereafter be modeled using a random effect model [26]. An approach for the binary outcome is to consider $Z_i = \text{logit}(p_i^{HT})$. If the design-based variance is denoted by V_i, the model is

$$Z_i \sim N(\mu_i, V_i)$$
$$\mu_i = S_i + \epsilon_i$$

where $\epsilon_i \sim_{iid} N(0, \sigma_\epsilon^2)$ for $i = 1 \cdots n$ and $S \in [1 \cdots 37]$ are the spatial locations (States of Nigeria) that are assigned a spatial distribution. Thus, an intrinsic conditional autoregressive (ICAR) prior, following the assumption that in general, events at neighboring locations are likely to be similar, was adopted. We implement the model using the `SUMMER` package in `R`. The package is useful for small area estimation using survey data collected with complex stratification designs [27]. The package builds on the `R-INLA` package using the BYM parameterisation for estimating the smoothed spatial effects [28]. We chose 90% credible intervals to quantify the significance of our estimates. To compare changes in events between the previous years and 2018 for each State, we subtracted the estimated prevalence between the years compared, while to determine the significance in the change, we estimated the difference in the credible

intervals such that whenever (a) the upper 90% credible interval value for earlier year is greater than the lower 90% credible interval value for 2018 or (b) the lower 90% for the earlier year is lower than the upper 90% for 2018, the difference is said to be not statistically significant.

Results & Discussion

Treatment Seeking Venues

Fig ?? presents the results for treatment seeking-behavior for diarrhea, indicating whether treatment was sought at a clinic (Fig ??A) or at a chemist/pharmacy (Fig ??B). For all the maps, States shaded with dots are places where the estimates are not significantly different from the national average. The findings reveal that about 20% or fewer of the children would be taken for treatment at a health facility among those who reside in Borno, Yobe, Taraba, Niger and Zamfara states while the estimates are not significant for the other states. This finding demonstrates the sub-optimal use of formal healthcare services for child health throughout the country and particularly among individuals in the northern fringes as revealed by previous studies [12, 29, 30]. Some authors attributed this to the prevailing cultural differences in perception of health and diseases, and the availability and distance to healthcare facilities [30].

In the case of chemist/pharmacy, a larger proportion of the children who live in Borno, Yobe, Bauchi and Kano states sought treatment for diarrhea at a chemist/pharmacy. Facing health service inacessibility and/or unaffordability the residents of these areas may turn to coping strategies such as ~~praying to God~~, use of traditional medicine and patronizing unaccredited chemists [31]. A systematic review reveals that in most low- and middle-income countries (LMIC) the majority of caregivers seek care for childhood illnesses but only a handful utilize formal care provider, with no care-seeking more common for diarrhea [32].

Fig 1: Model-based estimates of the breakdown of treatment seeking for early childhood diarrhea in Nigeria by venue and State for 2018.

Oral ~~Rehyrdation~~ Therapy

We estimated the national prevalence for the use of ORS/RHF received from any location to be 0.41 (90% CI: 0.31, 0.51) in 2008; 0.43 (90% CI: 0.32, 0.53) in 2013; and 0.45 (90% CI: 0.35, 0.55) in 2018, indicating a marginal increase of 4% over a ten-year period. Previous studies in different parts of the country reported 43.5% prevalence of ORS usage in Cross River State [33]; 49.5% in a Military Barrack in Ibadan [34], while Egbewale and colleagues [5] report 39.7% usage across Nigeria. When broken down by venue, findings from the 2018 data show that the recommended therapy was received by 24% (90% CI: 11%, 35%), 48% (90% CI: 31%, 64%) and 70% (90% CI: 54%, 83%) of children for whom treatment was sought at home, a chemist/pharmacy or at a health facility, respectively. The stratification by venue offers potentials for interventions through the venues, however, given that our data source provides information on venue choice amongst those who received ORS, this could confound venue preferencing with performance.

The State-level results presented in Fig **??** indicate that the proportion of children with diarrhea who received the recommended ORS/RHF in Kaduna, Plateau, Taraba, Gombe, Bauchi and Yobe States are lower than the national average (about 30% or lower) but higher among those residing in Borno, Jigawa, Kano and Nasarawa States; estimates for all the other States, including the FCT, are not significant. In Fig **??**B, we compute change in the proportion of children with diarrhea who received the recommended ORS/RHS therapy since 2008, with the aim of measuring State-specific progress over the ten-year period. The findings indicate significant positive change in Sokoto, Katsina, Kano, Jigawa, Bauchi, Borno and Nasarawa States but negative in Kaduna and Taraba, implying reduction in the proportion of children with diarrhea who received the recommended therapy in these two areas. Katsina, Kano and Bauchi States might have been impacted by the ECMPS program undertaken by CHAI [13] but this was not the case for the other states covered by the program; of particular concern is Kaduna where the estimates indicate a negative change that was significant. The dramatically high usage in Borno, a state affected by conflict since around 2009, could be attributed to focused interventions from the international community. The WHO trained a number of community resource persons on integrated community case

management of childhood illnesses who were deployed to internally displaced persons (IDP) camps to treat children with mild ailments such as diarrhea, malaria and pneumonia while those with serious conditions were to be referred to nearby health facilities [35]. There were also WHO-supported mobile health teams, a group of trained medical professionals who provide essential health services to vulnerable and displaced populations and host communities across states in the north east region [36].

Fig 2: Model-based estimates of the proportion of early childhood diarrhea cases receiving the recommended oral rehydration therapy in Nigeria by venue and State in 2018.

Furthermore, we compute the differences in proportion of children who received recommended ORS/RHF based on the venue where treatment was sought, considering each venue against all other venues (Fig **??**C, D, & E). The estimates suggest that the the use of ORS/RHS for treatment of diarrhea is modified by the place where treatment was sought. Specifically, with the exception of a few states namely, Borno, Nasarawa, Plateau, Ogun, Delta, Akwa Ibom and Rivers, where the estimates are not significant, the majority of the children from the other states for whom treatment was sought at a health facility would receive the recommended ORS/RHF. For those who sought treatment at a chemist/pharmacy, only Niger and Jigawa states show positive changes in the proportion of children who received the recommended therapy. Regarding the children for whom neither a health facility nor chemist/pharmacy was visited, the findings indicate negative change in the chances of receiving the recommended ORS/RHF in majority of the States, with no State having higher proportion in this category. Consequently, improvement in the use of ORS/RHF for treating diarrhea among children in Nigeria can be driven by care seeking at health facilities, similar to what was reported by Lam and colleagues [13].

Antibiotic Use

The national mean proportion of early childhood diarrhea cases treated with antibiotics was estimated to be 0.32 (90% CI: 0.22, 0.42) in 2008, 0.42 (90% CI: 0.31, 0.52) in 2013 and 0.27 (90% CI: 0.19, 0.36) in 2018. Venue specific estimates for 2018

were 0.13 (90% CI: 0.06, 0.24) for home, 0.35 (90% CI: 0.25, 0.53) for chemist/pharmacy and 0.35 (90% CI: 0.21, 0.51) for health sector. The State-specific estimates presented in Fig **??** show that a high proportion of the children who suffered from diarrhea and lived in Kaduna, Anambra, Niger, Taraba, Kano and Yobe (at least 70% in Kaduna and Anambra) would be treated with antibiotics. A study had reported that about 85% of children with acute diarrhea were prescribed antibiotics at healthcare facilities in Abakaliki, South East, Nigeria [20] while in another study, about half of the children used unprescribed antibiotics in Enugu [22]. These point to the widespread use of antibiotics for acute diarrhea in the country. The estimates are also significantly high but to a lesser extent for those in Adamawa, Plateau, Jigawa, Katsina, Kogi, Ondo and Osun states. On the change in use of antibiotics since 2008, the results show negative changes in Adamawa, Jigawa and Osun states but positive in Taraba. For the differences in the proportion of children who received antibiotics when care was sought at a health facility against other venues, the results indicate that children who reside in Bauchi, Plateau, Kano, Jigawa, Adamawa, Zamfara and the FCT are more likely to receive antibiotics when treated at a health facility; those who lived in Kebbi, Sokoto, Kwara, Kaduna, Kano, Gombe, Yobe, Benue, Imo and Abia States and visited a chemist/pharmacy would more likely to have been given antibiotics. In the majority of the States, those who neither visited a health facility nor a chemist/pharmacy were less likely to have been treated with antibiotics.

Fig 3: Model-based estimates of the proportion of early childhood diarrhea cases receiving antibiotics in Nigeria by venue and State in 2018.

Further Discussion

The treatment of childhood diarrhea in Nigeria has often been marred by poor knowledge of the use of appropriate low cost therapy [1]. A consequence is the increase in the number of episodes that young children incur in a year. There is a widespread use of antibiotics without diagnostic or epidemiological reasons to justify the use [20, 22]. Over the years, different interventions by local and international authorities to improve life-saving health services particularly to the most vulnerable

population have been contemplated but these have yielded mixed impact at different communities [13]. Thus, model-based estimates empower investigations at subnational levels, improving the understanding of local variations and trends and aiding targeted interventions. Geostatistical modeling endeavors in the Nigerian public health domain have therefore been of interest in recent years [10, 11].

The findings reveal that for most of the States, including those that have enjoyed specific interventions to improve diarrhea treatment, not much change has taken place over the ten-year period, with Kaduna showing a negative change in the use of ORS/RHF but positive for antibiotic use. However, in a State like Borno that has witnessed civil unrest for more than a decade, the findings indicate that a larger segment of the children with episode of diarrhea would benefit from the recommended therapy, a development that can be attributed to the deployment of trained personnel who serve as mobile health teams that provide essential health services to vulnerable and displaced populations [36]. For the majority of the States, the proportion of children with diarrhea for whom treatment was sought at a health facility was not significantly higher than the national average, although there is evidence that the use of ORS/RHF in Nigeria is ~~shaped by the~~ relative propensities of patients in different areas to seek care at health facilities. Consequently, a community-based approach as practiced in the conflict State of Borno can be considered in other parts of the country.

Conclusion

We have characterised spatial and temporal trends in the treatment seeking and treatment outcomes for episodes of early childhood diarrhea in Nigeria over the period 2008–2018 covered by three rounds of DHS surveys. While there have been substantial increases in the fraction of cases receiving treatment with recommended oral rehydration therapy at the national level, there has been substantial sub-national variation in these improvements, and antibiotics remain over-used in the treatment of diarrhea across Nigeria. While care seeking at a health facility is associated with an increased likelihood of receiving the recommended oral rehydration therapy, it is also associated with an increase likelihood of antibiotic treatment at a level equal to that of care seeking at a chemist/pharmacy.

Acknowledgments

Comments received on the work from members of the National Malaria Elimination Programme (NMEP) of Nigeria are appreciated.

References

1. Okafor IP, Akinyemi OT, Wika-Kobani BN, Olubodun T, Eze UT. Childhood diarrhoea: a cross-sectional survey on maternal knowledge, hygienic practices and use of oral zinc for home management in a Nigerian community. Pan African Medical Journal. 2022;42.

2. Teshale AB, Liyew AM, Tesema GA. Factors associated with zinc utilization for the management of diarrhea in under-five children in Ethiopia. BMC Public Health. 2020;20:1–7.

3. Walker CLF, Black RE. Zinc for the treatment of diarrhoea: effect on diarrhoea morbidity, mortality and incidence of future episodes. International journal of epidemiology. 2010;39(suppl_1):i63–i69.

4. Lukacik M, Thomas RL, Aranda JV. A meta-analysis of the effects of oral zinc in the treatment of acute and persistent diarrhea. Pediatrics. 2008;121(2):326–336.

5. Egbewale BE, Karlsson O, Sudfeld CR. Childhood Diarrhea Prevalence and Uptake of Oral Rehydration Solution and Zinc Treatment in Nigeria. Children. 2022;9(11):1722.

6. Ahinkorah BO, Aboagye RG, Seidu AA, Frimpong JB, Cadri A, Afaya A, et al. Prevalence and predictors of oral rehydration therapy, zinc, and other treatments for diarrhoea among children under-five in sub-Saharan Africa. Plos one. 2022;17(10):e0275495.

7. Wiens KE, Lindstedt PA, Blacker BF, Johnson KB, Baumann MM, Schaeffer LE, et al. Mapping geographical inequalities in oral rehydration therapy coverage in low-income and middle-income countries, 2000–17. The Lancet Global Health. 2020;8(8):e1038–e1060.

8. Ezezika O, Ragunathan A, El-Bakri Y, Barrett K. Barriers and facilitators to implementation of oral rehydration therapy in low-and middle-income countries: A systematic review. PLoS One. 2021;16(4):e0249638.

9. NPC, ICF. Nigeria Demographic and Health Survey 2018. Rockville, Maryland USA: National Population Commission, Federal Republic of Nigeria, Abuja, and ICF International; 2019.

10. Adebayo SB, Gayawan E, Heumann C, Seiler C. Joint modeling of Anaemia and Malaria in children under five in Nigeria. Spatial and spatio-temporal epidemiology. 2016;17:105–115.

11. Gayawan E, Orunmoluyi OS, Adegboye OA. Geostatistical patterns of comorbidity of diarrhea, acute respiratory infection, and stunting among under-five children in Nigeria. Mathematical Population Studies. 2022; p. 1–15.

12. Adeoti IG, Cavallaro FL. Determinants of care-seeking behaviour for fever, acute respiratory infection and diarrhoea among children under five in Nigeria. Plos one. 2022;17(9):e0273901.

13. Lam F, Abdulwahab A, Houdek J, Adekeye O, Abubakar M, Akinjeji A, et al. Program evaluation of an ORS and zinc scale-up program in 8 Nigerian states. Journal of global health. 2019;9(1).

14. Muslim OJ, Giwa A, Okoro NR, Giwa HB. Evaluation of mothers knowledge and attitudes of oral rehydration therapy in childhood diarrhoea. Bulletin of Pharmaceutical Research. 2018;8:trad023. doi:10.21276/bpr.2018.8.2.2.

15. Abiola A, Ndaman A, Idris S, Jiya N, Ibrahim M. Home management of childhood diarrhoea among mothers in Sokoto, Nigeria. Tropical Journal of Health Sciences. 2010;17(1).

16. Fagbamigbe AF, Juma J, Kariuki J. Barriers to utilisation of oral rehydration solution and zinc in managing diarrhoea among under-5 children in Oyo State, Nigeria. BMJ Paediatrics Open. 2022;6(1).

17. Ogundele OA, Bello OE, Ogundele T, Fagbemi AT, Fehintola FO, Osunmakinwa OO. Determinants of zinc utilisation in the management of

diarrhoea among under-five children in Nigeria: a population-based, cross-sectional study. Transactions of The Royal Society of Tropical Medicine and Hygiene. 2023; p. trad023.

18. Diniz-Santos DR, Silva LR, Silva N. Antibiotics for the empirical treatment of acute infectious diarrhea in children. Brazilian Journal of Infectious Diseases. 2006;10:217–227.

19. Organization WH, et al.. Antibiotic resistance: Multi-country public awareness survey; 2015.

20. Efunshile AM, Ezeanosike O, Nwangwu CC, König B, Jokelainen P, Robertson LJ. Apparent overuse of antibiotics in the management of watery diarrhoea in children in Abakaliki, Nigeria. BMC Infectious Diseases. 2019;19(1):1–7.

21. Auta A, Ogbonna BO, Adewuyi EO, Adeloye D, Strickland-Hodge B. Prevalence and factors associated with the use of antibiotics in non-bloody diarrhoea in children under 5 years of age in sub-Saharan Africa. Archives of Disease in Childhood. 2019;104(6):518–521.

22. Ekwochi U, Chinawa JM, Obi I, Obu HA, Agwu S. Use and/or misuse of antibiotics in management of diarrhea among children in Enugu, Southeast Nigeria. Journal of tropical pediatrics. 2013;59(4):314–316.

23. Ogbo PU, Aina BA, Aderemi-Williams RI. Management of acute diarrhea in children by community pharmacists in Lagos, Nigeria. Pharmacy practice. 2014;12(1).

24. FMOH, NPHCDA. Essential Childhood Medicines Scaleup Plan: 2012-2015. Abuja, Nigeria: Federal Ministry of Health, National Primary Health Care Development Agency; 2012.

25. Chen C, Wakefield J, Lumely T. The use of sampling weights in Bayesian hierarchical models for small area estimation. Spatial and spatio-temporal epidemiology. 2014;11:33–43.

26. Fay RE, Herriot RA. Estimates of income for small places: an application of James-Stein procedures to census data. Journal of the American Statistical Association. 1979;74(366a):269–277.

27. Martin B, Li Z, Hsiao Y, Godwin J, Wakefield J, Clark S. SUMMER: spatio-temporal under-five mortality methods for estimation. R package version 01 0. 2017;.

28. Bakka H, Rue H, Fuglstad GA, Riebler A, Bolin D, Illian J, et al. Spatial modeling with R-INLA: A review. Wiley Interdisciplinary Reviews: Computational Statistics. 2018;10(6):e1443.

29. Gayawan E. Spatial analysis of choice of place of delivery in Nigeria. Sexual & Reproductive Healthcare. 2014;5(2):59–67.

30. Abdulkadir MB, Abdulkadir ZA. A cross-sectional survey of parental care-seeking behavior for febrile illness among under-five children in Nigeria. Alexandria Journal of Medicine. 2017;53(1):85–91.

31. Adedayo A, Yusuf R. Health deprivation in rural settlements of Borno State, Nigeria. Journal of Geography and Geology. 2012;4(4):52.

32. Geldsetzer P, Williams TC, Kirolos A, Mitchell S, Ratcliffe LA, Kohli-Lynch MK, et al. The recognition of and care seeking behaviour for childhood illness in developing countries: a systematic review. PloS one. 2014;9(4):e93427.

33. Osonwa Kalu O, Eko Jimmy E, Ema S. Utilization of oral rehydration therapy in the management of diarrhea in children among nursing mothers in Odukpani local government area of cross River State, Nigeria. Am J Public Health Res. 2016;4(1):28–37.

34. Agbolade M, Dipeolu I, Ajuwon A. Knowledge and use of oral rehydration therapy among mothers of under-five children in a Military Barrack in Ibadan, Nigeria. African Journal of Biomedical Research. 2015;18(1):7–15.

35. WHO. Recently liberated communities in Borno state benefit from Integrated Management of Childhood Illnesses; 2016. Available from:

https://www.afro.who.int/news/
recently-liberated-communities-borno-state-benefit-integrated-management-childhood-il

36. WHO. WHO expands health services support for vulnerable populations in the northeast; 2023. Available from: https://www.afro.who.int/countries/nigeria/news/who-expands-health-services-support-vulnerable-populations-northeast.

Fig 1

Click here to access/download;Figure;TTFIG.png

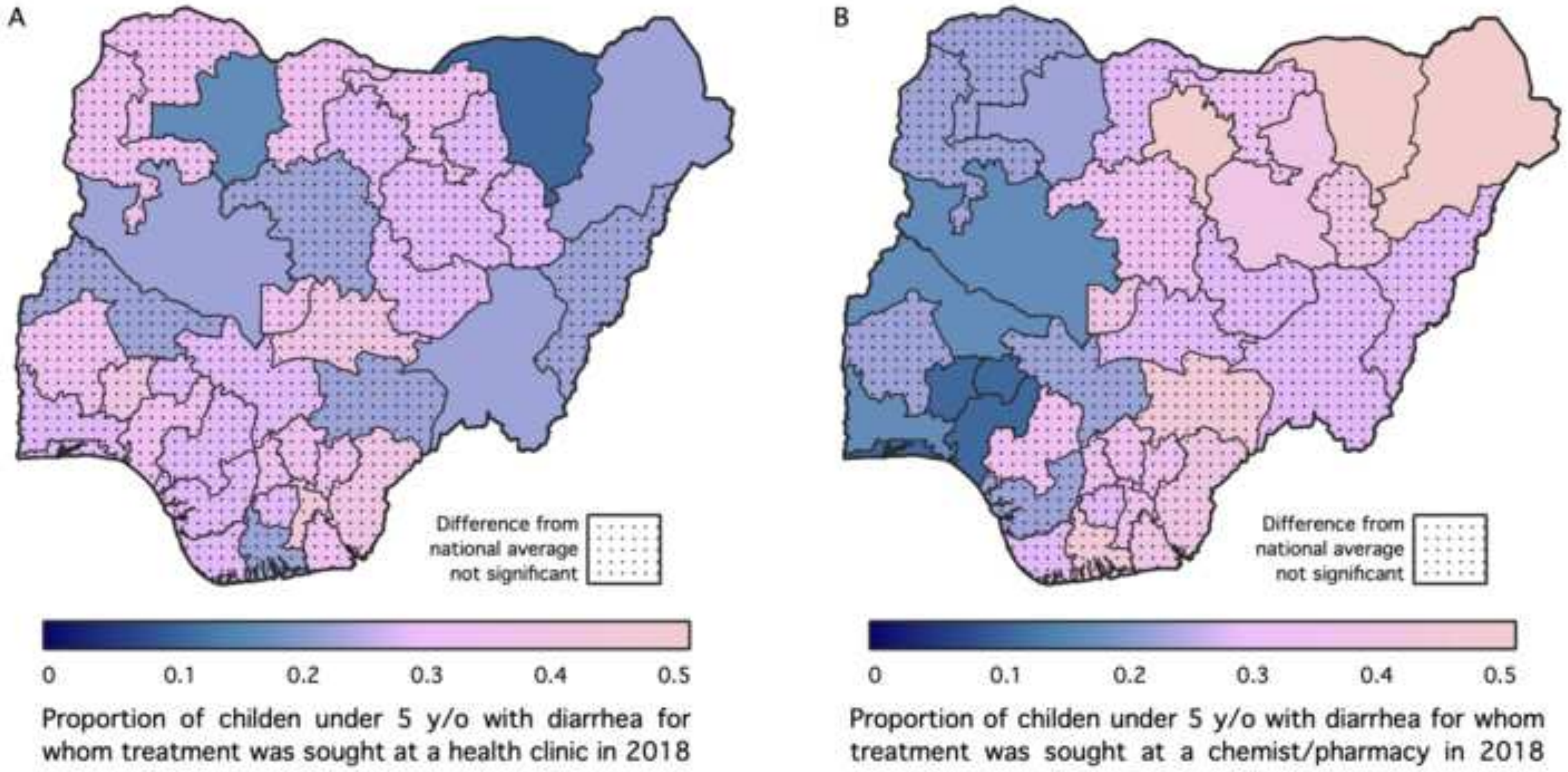

Fig 2

Click here to access/download;Figure;ORSFIG.png

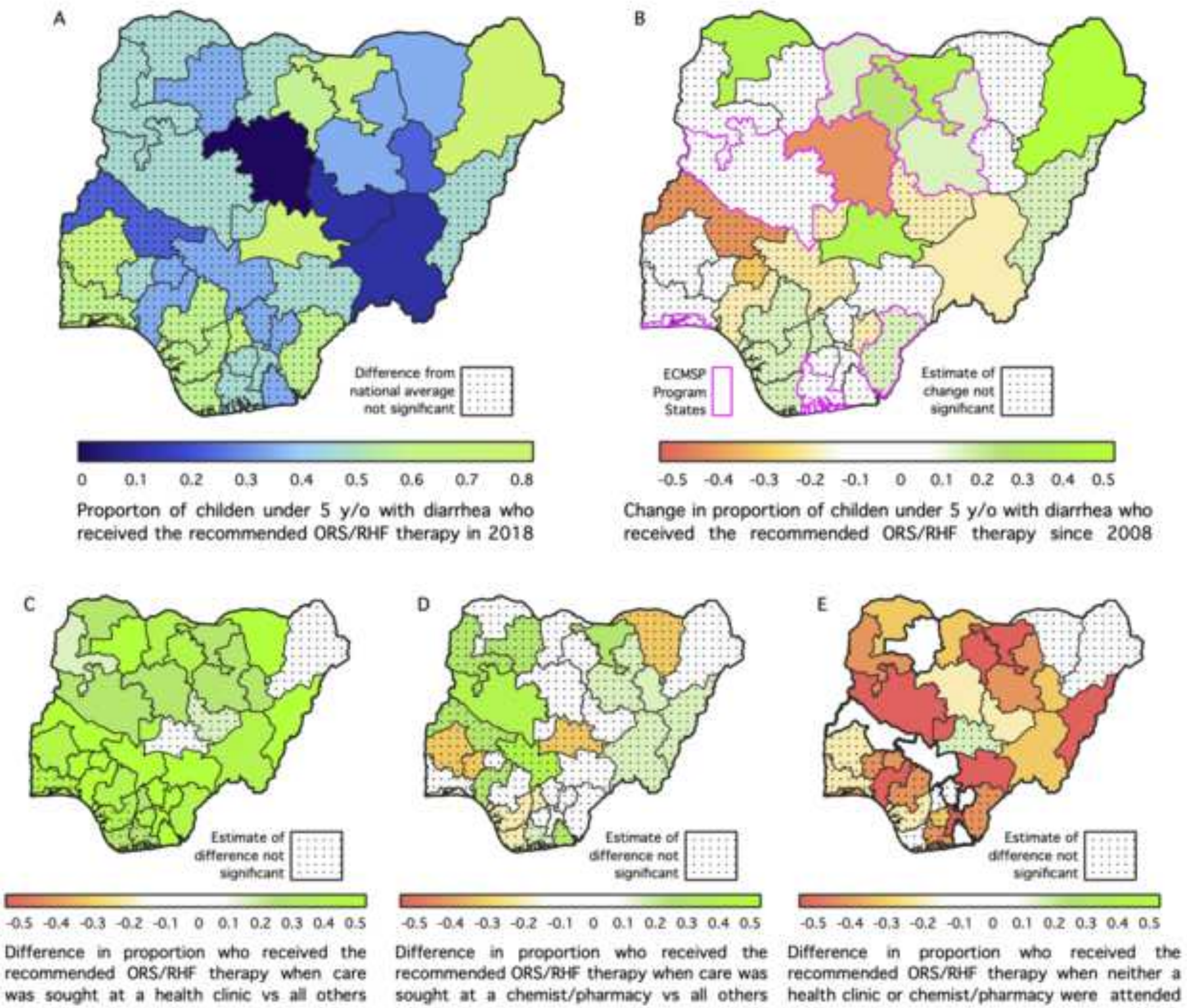

Fig 3

Click here to access/download;Figure;ABTFIG.png

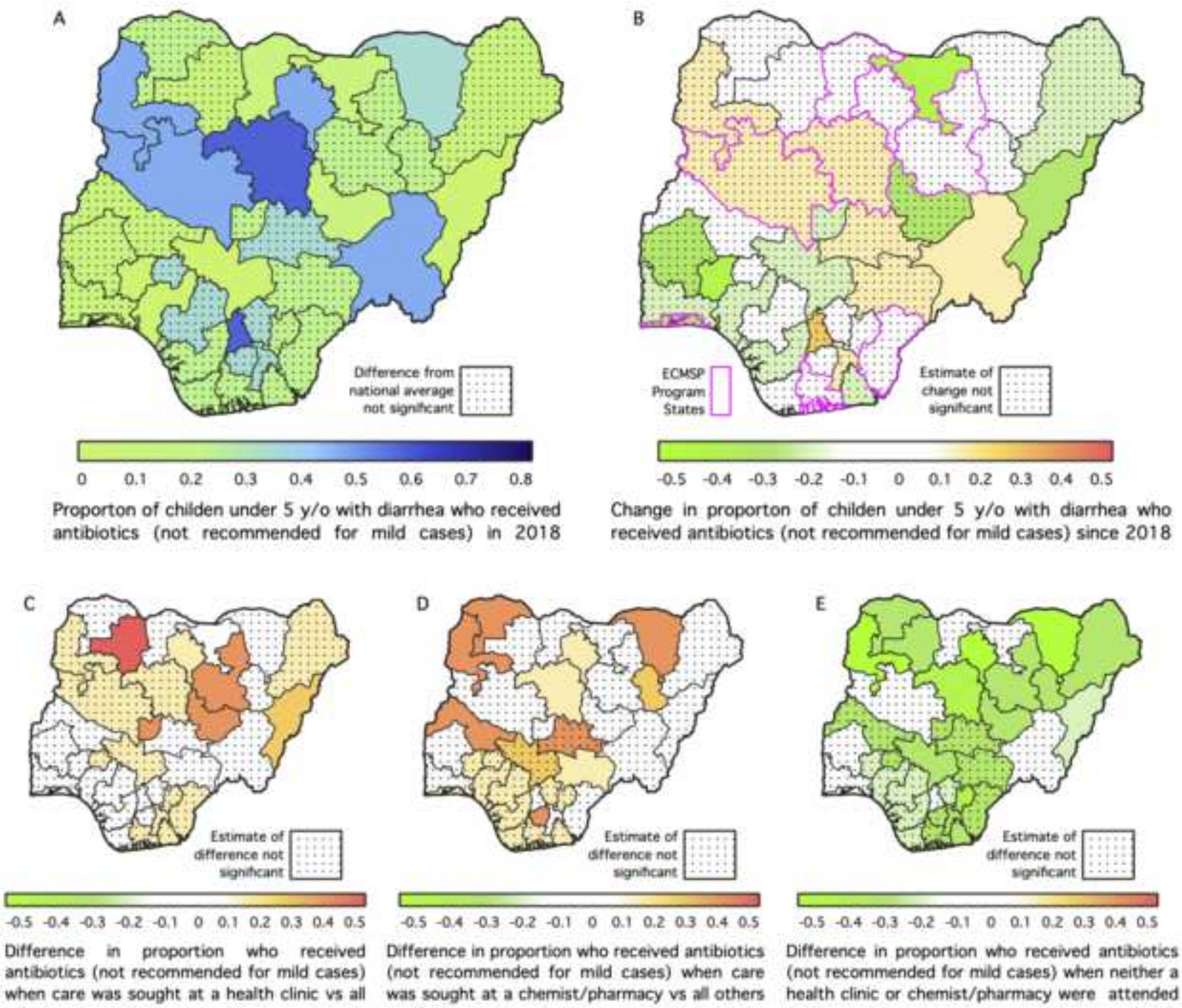

Supplement: Attachment — Submitted filename: PONE-D-23-41643_Reviewed.pdf [file pone.0303963.s002.pdf]
