# Supplementary material for: A situational assessment of treatments received for childhood diarrhea in the Federal Republic of Nigeria
Source: PLoS One. 2024 May 22;19(5):e0303963. doi: 10.1371/journal.pone.0303963 (PMC11111079; doi:10.1371/journal.pone.0303963)
Supplement: Attachment — Submitted filename: PONE-D-23-41643_comments.docx [file pone.0303963.s001.docx]

PONE-D-23-41643

Review: A situational assessment of treatments received for childhood

diarrhea in the Federal Republic of Nigeria

The study assessed for improvement in the case management of diarrhoea in children in Nigeria based on 3 Demographic health survey rounds conducted between 2008 and 2018. The study stratified changes in the proportion of children receiving oral rehydration therapy by venue of treatment and by state.

The study noted that there was significant improvement in the number of children who were seen in chemist or hospital facilities for diarrhoea who received oral rehydration therapy. However, the total number of children receiving oral rehydration therapy for diarrhoea in children was less than ideal due to state level variation in venue attendance and performance by the sites. The use of antibiotics, not particularly recommended in diarrhoea was rampant with negligible variation across venues. There was welcome reduction in the proportion of children with diarrhoea who received antibiotics in a few states.

Comments

The Study contributes important information which is useful for planning and evaluating interventions in the management of childhood diarrhoea.

The study is acceptable subject to the following major corrections.

1. The abstract section includes the following “Nationally, there have been substantial increases in the proportion of children under 5 years old with diarrhoea receiving the recommended oral rehydration therapy after seeking treatment at either a health clinic (0.57 [0.44{0.69; 95% CrI] in 2008; 0.70 [0.54{0.83] in 2018) or chemist/pharmacy (0.28 [0.17{0.42] in 2008; 0.48[0.31{0.64] in 2018).”

This was referred to in the results section lines 151-155, but the details of the change e.g 2008 values and the 2018 values should be included in the results and discussion section

2. Methods section: More comments on how the information on the history of antibiotic use for childhood diarrhoea was ascertained. How did mothers identify antibiotic pills or syrups- did they show the samples of what they had used, or show the child's prescription, was it simply based on recall, or was it assumed that if the child used any additional medication this was likely to be an antibiotic. More light should be thrown on this and some comments on the justification for and the limitations on the ascertainment of antibiotic use can be included in the discussion.
3. Conclusion: Lines 256-258 “While there have been substantial increases in the fraction of cases receiving treatment with recommended oral rehydration therapy at the national level” Line 148 Result section- says “results shows marginal increase (i.e 5%)”,… and does not to support the above conclusion.

Minor Corrections

1. Introduction:

The section is generally well written,

Line 38. “ Because it is rarely microbial caused….” microbial has been deleted and sentence replaced with ‘Because it is rarely caused by bacteria….” As most cases of diarhoea in children are indeed microbial, just that they are caused by viruses and not bacteria.

2. Typographical corrections e.g spelling of Rehydration- Line 145
3. Respective national values to be included as footnotes in relevant figures.
4. Other corrections as in the attached manuscript
