# Supplementary material for: A situational assessment of treatments received for childhood diarrhea in the Federal Republic of Nigeria
Source: PLoS One. 2024 May 22;19(5):e0303963. doi: 10.1371/journal.pone.0303963 (PMC11111079; doi:10.1371/journal.pone.0303963)
Supplement: Attachment — Submitted filename: PONE.docx [file pone.0303963.s003.docx]

**Responses to comments on PONE-D-23-41643**

**“A situational assessment of treatments received for childhood diarrhea in the Federal Republic of Nigeria”**

**Editor’s comments:**

The study is highly relevant and will guide funders and decision makers in setting formulating priorities in prevention, care and treatment of under-5 diarrhea. Please review and address the comments listed below in addition to reviewers' (1 and 2) comments.

Introduction:

- Consider making this section more concise focusing on the key objectives of the study.

We appreciate the comment and have restructured the last paragraph of the introduction, making the content more focused on the objectives of the study.

Methods

- Please explicitly indicate the study design. Even though DHS data was used, but it will be important for readers to understand the study design. We have written the Data section in a more explicit way that described the design and instrument used for data collection.
- Datasets: the language used sounds like the data was primarily collected for this study. Please adjust the language. We have adjusted the language to indicated that a survey team collected the data on behalf of The DHS Program.
- Will be good to briefly provide context about Nigeria geopolitical structure and also socioeconomic context of the 6 geopolitical zones (consider a map). This is important because some of the findings are closely related to these parameters. We have added a paragraph that indicates the geographical setting of Nigeria and supported this with a map.

Results & Discussion:

- Please consider splitting this section into “results” and “discussion” sections. The results can be concisely outlined for example in a table under relevant subsections or in any format the authors feel appropriate. We have now separated the “results” and “discussion” sections as suggested.
- Discuss findings chronologically in the discussion section. This has now been done.
- Subsection “further discussion”: This is confusing. Inline with the structure of the study, results and discussion, consider the following:  moving components in this subsection to relevant subsections; creating a subsection “change in trends” for most of the contents in the “further discussion” subsection. We have changed the “further discussion” section to a “discussion” one.
- Also ensure that all figures are presented in line with plosone figure guidelines: https://journals.plos.org/plosone/s/figures

Reviewers' comments:

Reviewer's Responses to Questions

**Comments to the Author**

1. Is the manuscript technically sound, and do the data support the conclusions?

The manuscript must describe a technically sound piece of scientific research with data that supports the conclusions. Experiments must have been conducted rigorously, with appropriate controls, replication, and sample sizes. The conclusions must be drawn appropriately based on the data presented.

Reviewer #1: Partly

Reviewer #2: Yes

2. Has the statistical analysis been performed appropriately and rigorously?

Reviewer #1: I Don't Know

Reviewer #2: Yes

3. Have the authors made all data underlying the findings in their manuscript fully available?

The [PLOS Data policy](http://www.plosone.org/static/policies.action#sharing) requires authors to make all data underlying the findings described in their manuscript fully available without restriction, with rare exception (please refer to the Data Availability Statement in the manuscript PDF file). The data should be provided as part of the manuscript or its supporting information, or deposited to a public repository. For example, in addition to summary statistics, the data points behind means, medians and variance measures should be available. If there are restrictions on publicly sharing data—e.g. participant privacy or use of data from a third party—those must be specified.

Reviewer #1: Yes

Reviewer #2: Yes

4. Is the manuscript presented in an intelligible fashion and written in standard English?

PLOS ONE does not copyedit accepted manuscripts, so the language in submitted articles must be clear, correct, and unambiguous. Any typographical or grammatical errors should be corrected at revision, so please note any specific errors here.

Reviewer #1: Yes

Reviewer #2: Yes

5. Review Comments to the Author

Please use the space provided to explain your answers to the questions above. You may also include additional comments for the author, including concerns about dual publication, research ethics, or publication ethics. (Please upload your review as an attachment if it exceeds 20,000 characters)

Reviewer #1: Review: A situational assessment of treatments received for childhood
diarrhea in the Federal Republic of Nigeria
The study assessed for improvement in the case management of diarrhoea in children in Nigeria based on 3 Demographic health survey rounds conducted between 2008 and 2018. The study stratified changes in the proportion of children receiving oral rehydration therapy by venue of treatment and by state.
The study noted that there was significant improvement in the number of children who were seen in chemist or hospital facilities for diarrhoea who received oral rehydration therapy. However, the total number of children receiving oral rehydration therapy for diarrhoea in children was less than ideal due to state level variation in venue attendance and performance by the sites. The use of antibiotics, not particularly recommended in diarrhoea was rampant with negligible variation across venues. There was welcome reduction in the proportion of children with diarrhoea who received antibiotics in a few states.

We appreciate this detailed review.
Comments
The Study contributes important information which is useful for planning and evaluating interventions in the management of childhood diarrhoea.
The study is acceptable subject to the following major corrections.
1. The abstract section includes the following “Nationally, there have been substantial increases in the proportion of children under 5 years old with diarrhoea receiving the recommended oral rehydration therapy after seeking treatment at either a health clinic (0.57 [0.44{0.69; 95% CrI] in 2008; 0.70 [0.54{0.83] in 2018) or chemist/pharmacy (0.28 [0.17{0.42] in 2008; 0.48[0.31{0.64] in 2018).”
This was referred to in the results section lines 151-155, but the details of the change e.g 2008 values and the 2018 values should be included in the results and discussion section.

We have now presented a Table that includes all these estimates.

2. Methods section: More comments on how the information on the history of antibiotic use for childhood diarrhoea was ascertained. How did mothers identify antibiotic pills or syrups- did they show the samples of what they had used, or show the child's prescription, was it simply based on recall, or was it assumed that if the child used any additional medication this was likely to be an antibiotic. More light should be thrown on this and some comments on the justification for and the limitations on the ascertainment of antibiotic use can be included in the discussion.
We appreciate this insightful comment and have included this in the limitation of the study as a last paragraph before the concluding section. To our knowledge, the information was obtained based on recall and we admit that it could be subject to recall error and the ability to recognize that an antibiotic was administered.
3. Conclusion: Lines 256-258 “While there have been substantial increases in the fraction of cases receiving treatment with recommended oral rehydration therapy at the national level” Line 148 Result section- says “results shows marginal increase (i.e 5%)”,… and does not to support the above conclusion. We have made changes to this, thank you.

Minor Corrections
1. Introduction:
The section is generally well written,
Line 38. “ Because it is rarely microbial caused….” microbial has been deleted and sentence replaced with ‘Because it is rarely caused by bacteria….” As most cases of diarhoea in children are indeed microbial, just that they are caused by viruses and not bacteria. We have corrected this.
2. Typographical corrections e.g spelling of Rehydration- Line 145 We have corrected this.


3. Respective national values to be included as footnotes in relevant figures. We have now included a Table that contains the national values.

4. Other corrections as in the attached manuscript. They were well noted. Thank you for carefully reading through and providing the comments.

Reviewer #2: This is a very well written manuscript. The technical aspects are very sound, and it makes for easy reading. It does not appear to have any ethical concerns. There are a few typographical/spelling errors but not of any significant magnitude.

We appreciate the comments. We have carefully read through and correct the spelling errors throughout the manuscript.

6. PLOS authors have the option to publish the peer review history of their article ([what does this mean?](https://journals.plos.org/plosone/s/editorial-and-peer-review-process#loc-peer-review-history)). If published, this will include your full peer review and any attached files.


If you choose “no”, your identity will remain anonymous but your review may still be made public.


**Do you want your identity to be public for this peer review?** For information about this choice, including consent withdrawal, please see our [Privacy Policy](https://www.plos.org/privacy-policy).

Reviewer #1: No

Reviewer #2: **Yes:** Patience Ngozi Obiagwu
